# Sperm DNA Fragmentation in Native Semen: A Reflection of Apoptotic and Non-Viable Spermatozoa and Its Implications for Assisted Reproduction

**DOI:** 10.3390/ijms27177854

**Published:** 2026-09-02

**Authors:** András Balló, Natália Honétzy, Gábor Máté

**Affiliations:** 1 Tapolca Institute, Dunamenti REK Reproduction Center Ltd., 8300 Tapolca, Hungary; ballo.andras@pte.hu; 2 National Laboratory on Human Reproduction, University of Pécs, 7624 Pécs, Hungary; 3 Urology Clinic, University of Pécs Clinical Centre, 7621 Pécs, Hungary; 4 Institute of Biology, University of Pécs, 7624 Pécs, Hungary; nata.honetzy@gmail.com; 5 Department of Analytical Biochemistry, Institute of Biochemistry and Medical Chemistry, University of Pécs Medical School, 7624 Pécs, Hungary

**Keywords:** sperm DNA fragmentation (SDF), apoptosis, male infertility, sperm processing, assisted reproduction, motility, vitality, DNA fragmentation index (DFI), sperm chromatin structure assay (SCSA), ZyMōt

## Abstract

Sperm DNA fragmentation (SDF) is widely used as a biomarker of male fertility, although its predictive value for assisted reproductive technology (ART) outcomes remains controversial. We hypothesised that this discrepancy reflects the inclusion of non-viable spermatozoa in conventional SDF assessment of native semen. We retrospectively analysed semen samples from 1394 men to evaluate associations between DNA fragmentation index (DFI), sperm vitality, motility, and concentration. In addition, the viability-gated sperm chromatin structure assay (SCSA) was performed in a prospective cohort of 11 samples, and DFI was assessed before and after density gradient centrifugation, swim-up, microfluidic selection, and magnetic-activated cell sorting. Native semen DFI showed significant negative correlations with sperm vitality, motility, and concentration. Viability-gated analysis demonstrated a 3.5-fold lower mean DFI in viable spermatozoa than in the total ejaculate (11.27% vs. 39.64%, *p* < 0.001). All sperm preparation methods significantly reduced DFI while enriching motile and viable spermatozoa. These findings suggest that a substantial proportion of SDF detected in native semen originates from non-viable spermatozoa and that native semen DFI may not fully represent the DNA integrity of the fertilisation-competent sperm fraction, providing a potential biological explanation for its limited predictive value in ART.

## 1. Introduction

Infertility and the male factor

Infertility affects approximately 8–12% of couples of reproductive age worldwide; male factors are either solely responsible or contribute in up to 50% of all cases [1,2,3]. Infertility is defined by the WHO as the failure of a couple to achieve a clinical pregnancy after 12 months or more of regular unprotected sexual intercourse [1]. Recent Global Burden of Disease 2021 estimates indicated that 55 million men worldwide were living with infertility [2]. The laboratory evaluation of male infertility is centred on standardised semen analysis according to the WHO Laboratory Manual, including sperm concentration, motility and morphology, with sperm vitality providing an assessment of membrane integrity when indicated [1]. Although conventional semen parameters provide a useful clinical overview, they offer limited information on sperm functional and genetic integrity. A substantial proportion of male infertility cases remain unexplained despite apparently normal semen parameters—so-called idiopathic male infertility [3]. This diagnostic gap has driven interest in supplementary sperm function tests, among which sperm DNA fragmentation (SDF) has attracted considerable clinical and scientific attention [4,5,6].

Sperm DNA fragmentation: definition and measurement

SDF refers to the presence of single- or double-strand breaks within the sperm nuclear DNA. A number of techniques have been developed to quantify SDF. These include the sperm chromatin structure assay (SCSA) [7], the Sperm Chromatin Dispersion (SCD) test [8], the terminal deoxynucleotidyl transferase dUTP nick end labelling (TUNEL) assay [9], and the single-cell gel electrophoresis (Comet) assay [10], as summarised in the systematic review by Cissen et al. [11]. Each method interrogates DNA damage using a different analytical principle and may yield different clinical thresholds, contributing to heterogeneity among published studies [11,12]. SDF testing has been proposed as a clinically meaningful adjunct to conventional semen analysis in selected infertility scenarios [13]. However, the extent to which an elevated DNA fragmentation index (DFI) measured in native, unprocessed semen predicts outcomes after assisted reproductive technology (ART) remains controversial [11,12]. Here, we critically evaluate this question in light of the biological origins of SDF and the obligatory sperm-processing steps that precede fertilisation in ART. The term native semen is used consistently to denote the unprocessed ejaculate before any sperm-selection procedure.

Mechanisms of sperm DNA fragmentation: the central role of apoptosis

Three principal mechanisms have been proposed for the origin of SDF: (i) abortive apoptosis during spermatogenesis; (ii) defective chromatin remodelling during spermiogenesis; and (iii) oxidative stress-induced DNA damage during epididymal transit and after ejaculation [14]. Of these, abortive apoptosis is most relevant to the present study. During normal spermatogenesis, apoptosis eliminates excess or genetically compromised germ cells via both intrinsic (mitochondria-dependent) and extrinsic (Fas-mediated) pathways [15]. When this process is initiated but fails to eliminate the cell entirely—so-called abortive apoptosis—the spermatozoon that reaches the ejaculate carries molecular hallmarks of programmed cell death: active caspases, externalised phosphatidylserine, altered mitochondrial membrane potential, and fragmented DNA [15,16]. Muratori et al. reported that active caspases and cleaved poly(ADP-ribose) polymerase (cPARP) co-occurred with SDF in 82.6% and 53.5% of DNA-fragmented spermatozoa, respectively, supporting the conclusion that apoptosis is a principal pathway leading to sperm DNA strand breaks [14]. Oxidative stress contributes secondarily, particularly in viable cells, while defective histone-to-protamine exchange further increases chromatin vulnerability [14].

Relationship between sperm vitality, motility, and DNA fragmentation

If SDF in native semen is strongly influenced by the non-viable sperm fraction, an inverse association between membrane integrity (vitality), motility, and DFI would be expected. Motility requires an intact cellular function and is generally associated with viability, although viable spermatozoa may also be immotile. Góngora et al., analysing 1159 ejaculates, reported a significant positive association between the proportions of membrane-compromised and immotile spermatozoa [17]. Several studies have also shown inverse associations between DFI and progressive motility, vitality, and sperm survival [4,18,19,20,21]. Thus, samples containing a larger non-viable fraction may show a higher population-level DFI, while this relationship does not exclude DNA fragmentation within viable spermatozoa.

Sperm preparation in ART and its effect on SDF

In standard assisted reproductive technology (ART) practice, fertilisation is not performed using native, unprocessed semen. Procedures such as intrauterine insemination (IUI), in vitro fertilisation (IVF), and intracytoplasmic sperm injection (ICSI) require prior sperm preparation. Principal preparation techniques—including swim-up, density gradient centrifugation (DGC), magnetic-activated cell sorting (MACS), and microfluidic selection—enrich motile and viable spermatozoa and generally produce fractions with a lower DFI than native semen [22,23,24,25]. Swim-up relies on autonomous migration of progressively motile cells, thereby excluding most immotile and non-viable cells. DGC separates cells by density, recovering a motile-enriched sperm fraction [22,23]. MACS removes phosphatidylserine-positive apoptotic spermatozoa through annexin V binding [24]. Microfluidic devices select actively migrating spermatozoa without centrifugation, thereby limiting centrifugation-associated oxidative stress [25].

Clinical paradox: High native DFI is not consistently associated with reduced ART pregnancy rates.

Multiple meta-analyses have not consistently demonstrated a significant association between high native semen DFI and impaired clinical pregnancy or live birth rates in IVF or ICSI cycles. Chen et al. [12], in a meta-analysis of 11 cohort studies, found no significant effect of DFI on IVF pregnancy rates (RR 0.83, 95% CI 0.57–1.21, *p* = 0.32), live birth rates, or any ICSI outcome measure. Cissen et al. [11] reported limited to very low specificity of SDF tests for predicting ongoing ART pregnancy. Zini et al. [26] concluded that sperm DNA damage was not significantly associated with ART pregnancy rates overall. This paradox is precisely what would be expected if sperm processing systematically eliminates the apoptotic, SDF-bearing fraction before fertilisation—making the native DFI measurement less directly relevant to the eventual outcome. The present study was designed to test this hypothesis directly using prospective clinical laboratory data.

The aims of the present study were: (i) to investigate the relationship between DFI and standard sperm functional parameters (concentration, total motility, progressive motility, and vitality) in a large retrospective clinical dataset; (ii) to directly compare DFI in the total native ejaculate versus the viable-gated sperm subpopulation within the same sample using flow cytometry; (iii) to quantify the effect of four standard ART sperm-preparation techniques (swim-up, DGC, MACS, and ZyMōt microfluidic chip) on motility, vitality, and DFI; and (iv) to evaluate whether the observed relationships collectively support the hypothesis that native semen DFI is primarily a measure of the apoptotic and non-viable sperm burden in the ejaculate, with limited predictive utility for ART outcomes.

## 2. Results

### 2.1. DFI Correlates Inversely with Sperm Functional Parameters in 1394 Samples

**Figure 1:**
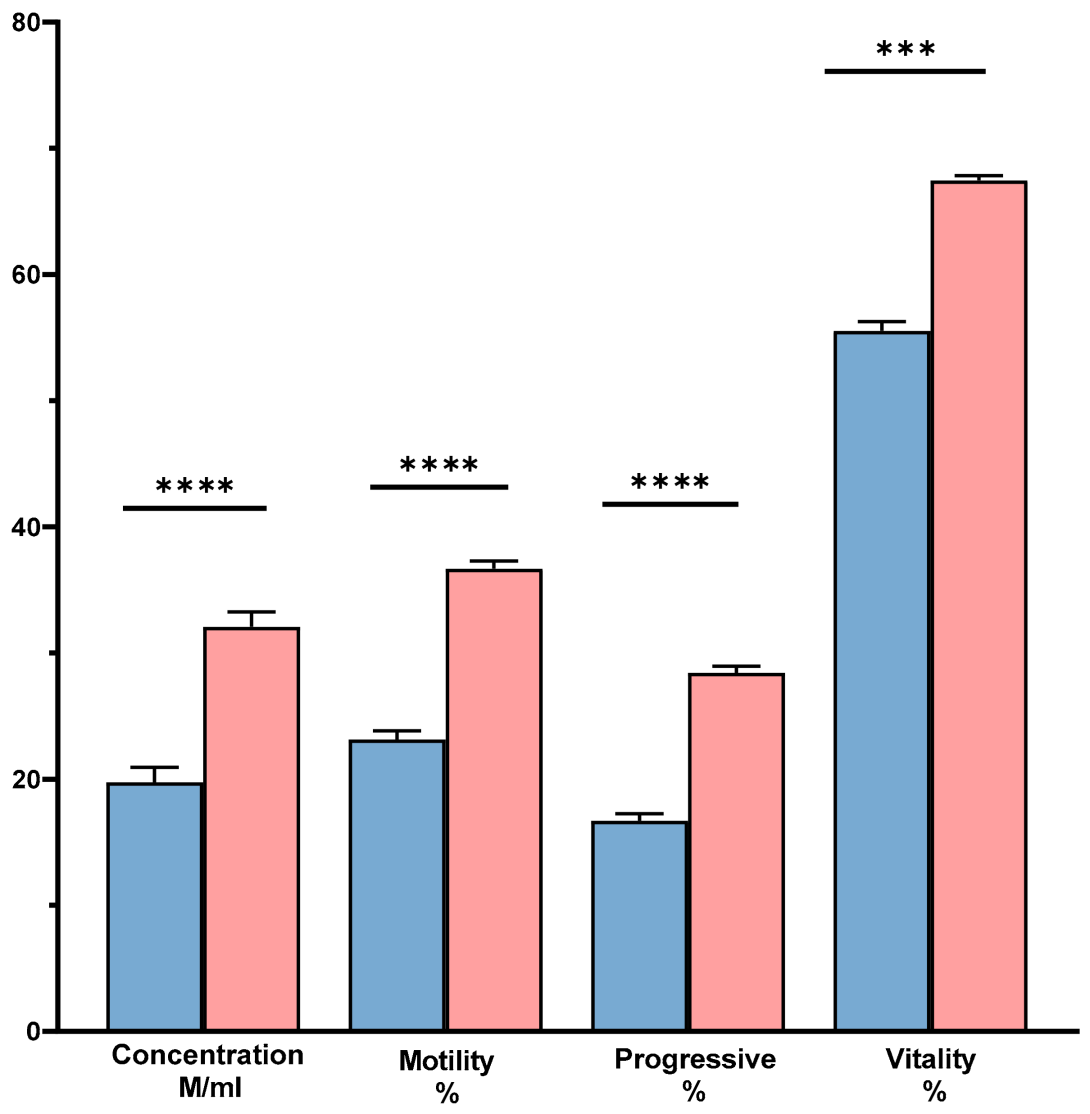
Retrospective analysis of 1394 semen samples. Blue columns indicate samples with DFI > 25%, and pink columns indicate samples with DFI < 25%. All investigated parameters differed significantly between groups. *** *p* < 0.001, **** *p* < 0.0001.

A retrospective analysis was performed using data from 1394 consecutive semen analyses that met the inclusion criterion that concentration, total motility, progressive motility, vitality, and DFI had all been measured. Samples were stratified by DFI threshold, and the association between DFI stratum and all the measured semen parameters was assessed (Kruskal–Wallis omnibus: H = 5935; *p* < 0.0001; df = 11). Samples with DFI < 25% had significantly higher sperm concentration, total motility, progressive motility, and vitality than samples with DFI > 25% (all pairwise comparisons *p* < 0.0001 after Dunn post hoc correction). Conversely, samples with DFI > 25% displayed substantially impaired motility, vitality, and concentration (Figure 1). These findings demonstrate a strong sample-level association between elevated native-semen DFI and impaired conventional semen parameters.

### 2.2. DFI in Viable-Gated Spermatozoa Is Dramatically Lower than in the Total Native Ejaculate

**Figure 2:**
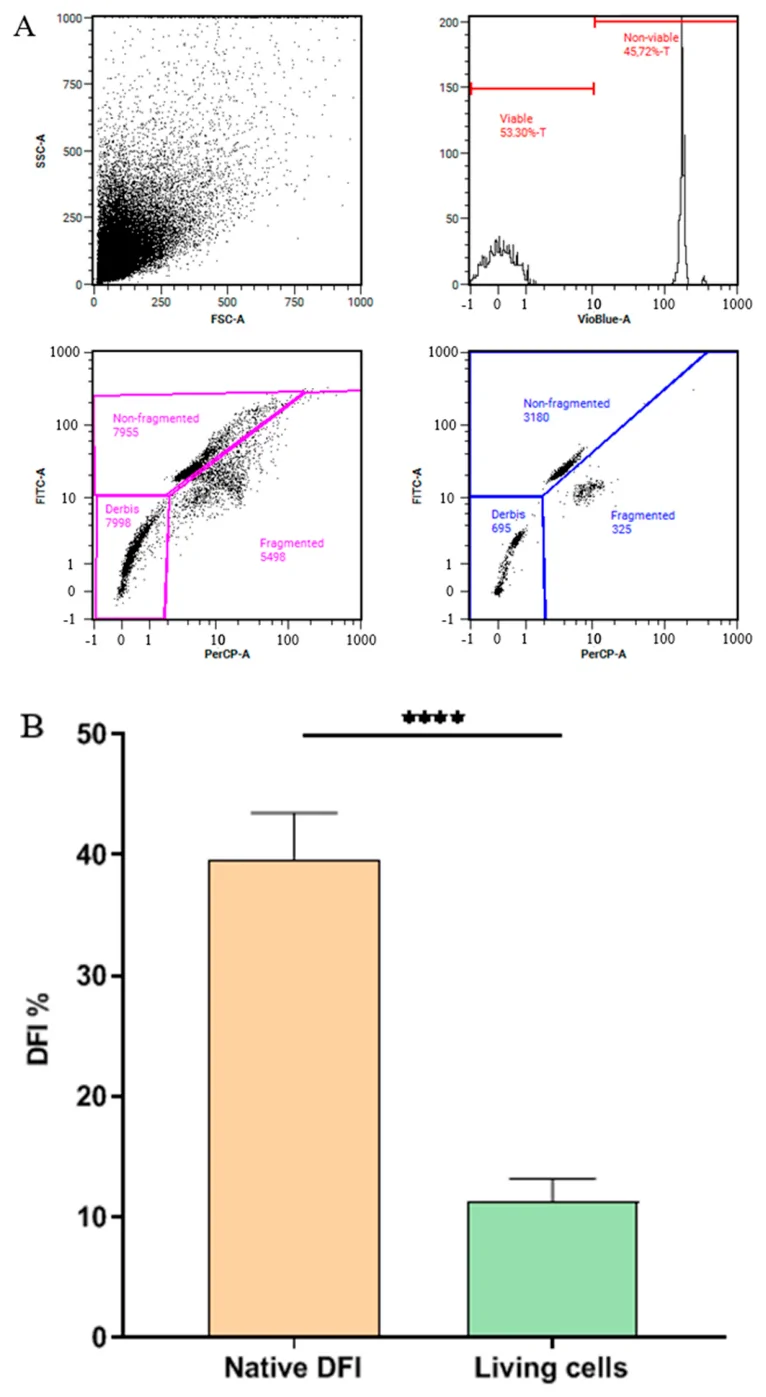
Determination of DFI on native ejaculate and viable-gated cells. (**A**) Representative flow cytometry dot plot and corresponding histogram showing gating of the total (native) sperm population versus the Viobility-negative (viable) subpopulation, with red (fragmented, single-stranded DNA) versus green (intact, double-stranded DNA) fluorescence used to derive DFI in each gate. (**B**) Paired comparison of DFI in the total ejaculate versus the viable-gated subpopulation across the 11 samples analysed. Viable cells showed significantly lower DFI. **** *p* < 0.0001.

In 11 paired samples in which DFI was measured simultaneously in the total native-semen sperm population and in the viable-gated subpopulation (Viobility^−^), the mean DFI was 39.64% in the total population and 11.27% in the viable-gated fraction (Wilcoxon matched-pairs signed-rank test, *p* < 0.0001; *n* = 11) (Figure 2). This 3.5-fold difference was observed by flow-cytometric gating without physical sperm selection. The result indicates that, in this small prospective cohort, a substantial proportion of the DNA-fragmentation signal in native semen was contributed by non-viable spermatozoa. The viable subpopulation nevertheless retained measurable DFI, emphasising that DNA damage is not restricted exclusively to non-viable cells.

### 2.3. All ART Preparation Methods Significantly Reduce DFI by Enriching Motile, Viable Spermatozoa

**Figure 3:**
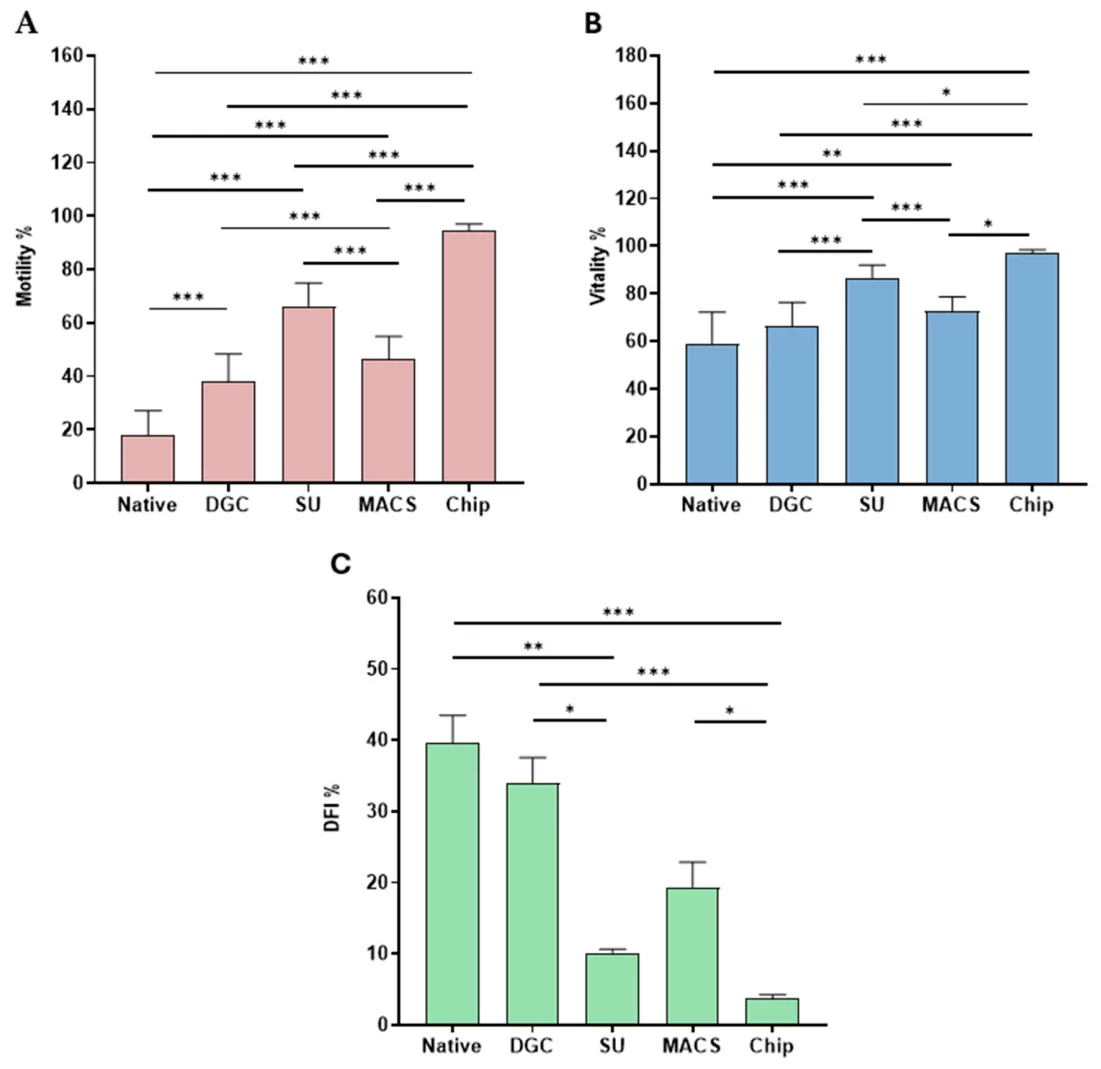
Motility (**A**), vitality (**B**), and DFI (**C**) after different preparation techniques (Native: native ejaculate; DGC: density gradient centrifugation; SU: swim-up; MACS: magnetic-activated cell sorting; and Chip: Zymōt chip separations). Different sperm processing techniques effectively improve motility and vitality, reducing DFI in parallel. * *p* < 0.05, ** *p* < 0.01, *** *p* < 0.001.

All four preparation techniques significantly reduced DFI compared with the native ejaculate, while simultaneously improving motility and vitality (Table 1 and Table 2). The omnibus Kruskal–Wallis test for DFI across groups was significant (H = 41.81; *p* < 0.0001; df = 4), as were the ANOVA results for vitality (F = 36.71; *p* < 0.0001; df = 4) and total motility (F = 134.6; *p* < 0.0001; df = 4). The greatest absolute DFI reduction was achieved by the ZyMōt microfluidic chip (from 39.64% in native semen to 3.68% post-chip), which also produced the highest post-preparation motility (94.36 ± 2.62%) and vitality (96.90 ± 1.42%). Swim-up achieved the second lowest post-preparation DFI (10.14 ± 1.61%), accompanied by improvements in motility (66.29 ± 8.60%) and vitality (86.39 ± 5.62%). MACS reduced DFI to 19.38 ± 11.55%, with intermediate motility and vitality gains. DGC produced the smallest DFI reduction (33.90 ± 12.11%), while still significantly improving motility and vitality over the native sample (Table 1 and Figure 3). The rank order of DFI reduction efficiency—ZyMōt chip > swim-up > MACS > DGC—closely mirrors the degree of motility-dependent selection applied by each method and is consistent with the hypothesis that motility and viability are the primary determinants of DNA integrity in the selected sperm subpopulation.

**Table 1:** Sperm parameters before (native) and after each preparation technique. Data are means ± SD.

Parameter	Native	DGC	Swim-up	MACS	ZyMōt Chip
Vitality (%)	58.65 ± 13.64	66.21 ± 10.11	86.39 ± 5.62	72.54 ± 6.09	96.90 ± 1.42
Total motility (%)	17.96 ± 9.22	37.95 ± 10.38	66.29 ± 8.60	46.33 ± 8.58	94.36 ± 2.62
DFI (%)	39.64 ± 12.67	33.90 ± 12.11	10.14 ± 1.61	19.38 ± 11.55	3.68 ± 1.84

**Table 2:** *p*-values of the dataset in Table 1.

Comparison	Motility	Vitality	DFI%
Native vs. DGC	*p* < 0.0001	*p* = 0.2392	*p* > 0.9999
Native vs. Swim-up	*p* < 0.0001	*p* < 0.0001	*p* = 0.0033
Native vs. MACS	*p* < 0.0001	*p* = 0.003	*p* = 0.1721
Native vs. ZyMōt	*p* < 0.0001	*p* < 0.0001	*p* < 0.0001
DGC vs. Swim-up	*p* < 0.0001	*p* < 0.0001	*p* = 0.0294
DGC vs. MACS	*p* = 0.1443	*p* = 0.4118	*p* = 0.7784
DGC vs. ZyMōt	*p* < 0.0001	*p* < 0.0001	*p* < 0.0001
Swim-up vs. MACS	*p* < 0.0001	*p* = 0.0032	*p* > 0.9999
Swim-up vs. ZyMōt	*p* < 0.0001	*p* = 0.0378	*p* = 0.3977
MACS vs. ZyMōt	*p* < 0.0001	*p* = 0.0001	*p* = 0.0109

## 3. Discussion

The principal finding of this study is that DFI measured in native semen is closely associated with conventional indicators of sperm quality and is substantially lower when analysis is restricted to viable spermatozoa. In the paired flow-cytometric cohort, viable-gated spermatozoa had a mean DFI of 11.27% compared with 39.64% in the total native-semen population. Because the comparison was obtained by gating within the same samples, without a physical sperm-selection step, it supports the interpretation that non-viable spermatozoa contribute substantially to the DFI measured at the whole-sample level. This interpretation is biologically plausible because apoptosis and related cell-death pathways are established contributors to sperm DNA damage [14,27]. Muratori et al. reported co-occurrence of active caspases and cPARP with SDF in DNA-fragmented spermatozoa [14], while Omran et al. similarly demonstrated co-detection of apoptotic markers and SDF in sperm from infertile men [27]. Importantly, our data do not imply that DNA fragmentation is absent from viable spermatozoa; rather, they indicate that viability status is an important determinant of population-level DFI.

The large retrospective dataset provides complementary evidence (Figure 1). Across 1394 eligible analyses, higher DFI was associated with lower sperm concentration, total motility, progressive motility, and vitality. These sample-level associations are consistent with previous reports describing a strong inverse relationship between sperm viability and DFI [4,20] and negative associations between DFI and other semen-quality measures [21]. The present data therefore support a close relationship between DFI and the overall quality of the semen sample, while they do not by themselves establish a causal pathway.

A clinically important consequence is that total DFI and DFI within viable spermatozoa are not necessarily equivalent biological measurements. Muratori et al. used LiveTUNEL flow cytometry to evaluate viable and non-viable sperm subpopulations and found that total, viable, and non-viable SDF could all discriminate fertile from subfertile men, but with different relationships to age and semen-quality variables [28]. In their study, viable SDF appeared to represent a distinct component of DNA damage that was less tightly coupled to conventional semen quality. This distinction is relevant to ART because the spermatozoa ultimately used for fertilisation are selected from the viable, motile fraction rather than randomly from the native sample.

The same concept is supported by the cross-sectional study of Deng et al., in which viable SDF showed better diagnostic accuracy for male infertility than total SDF (AUC 0.81 vs. 0.74) [29]. Together with our paired analysis, these findings suggest that viability-gated or post-selection measurements may provide information that is not captured by total native-semen DFI alone. Nevertheless, the small size of our viability-gated cohort (*n* = 11) requires caution, and larger studies are needed before a change in routine clinical testing can be recommended (Figure 2).

Recent evidence continues to support the view that native, unselected DFI is a poor predictor of downstream ART success, even as its diagnostic value for infertility per se is confirmed. Zurera-Egea et al., comparing total SDF by TUNEL in 20 fertile donors and 40 infertile patients, found that SDF was significantly higher in the infertile group and correlated negatively with count, motility, and morphology, confirming its diagnostic utility for infertility (AUC 0.72), yet without directly addressing predictive value for ART outcome [6]. By contrast, in a large retrospective analysis of 870 single-blastocyst ICSI cycles, Machałowski et al. found that elevated SDF impaired fertilisation rate and blastocyst development but was not predictive of clinical pregnancy, indicating that its impact is confined to the early, pre-selection stages of the ART process [30]. Similarly, Jiang et al., analysing DFI thresholds ranging from 15% to 30% across the literature, concluded that native DFI has limited effectiveness in predicting embryo quality in ART treatments, further reinforcing the distinction between DFI as a marker of male infertility and DFI as a predictor of the ART outcome achieved with a selected, prepared sperm sample [31].

The sperm-preparation experiments further extend this observation. All four methods reduced DFI while increasing motility and vitality, but the magnitude of change differed among techniques. The lowest post-preparation DFI values were observed after the ZyMōt microfluidic chip and swim-up, both of which depend strongly on autonomous sperm motility. Previous studies likewise reported lower DFI after swim-up than after DGC [32] and substantial reductions after combined DGC/swim-up processing [33]. These findings are consistent with enrichment of a motile, viable subpopulation with lower DNA fragmentation, although method-specific effects—including centrifugation-associated oxidative stress—may also contribute [23,25].

MACS produced an intermediate reduction in DFI, which is consistent with its direct removal of annexin V-binding spermatozoa, whereas DGC produced the smallest reduction among the four methods evaluated. The present study was not designed or powered as a head-to-head clinical comparison of sperm-selection methods, and therefore, the observed ranking should be interpreted descriptively rather than as evidence of clinical superiority. In particular, the absence of reproductive outcome data prevents conclusions regarding whether the lower post-processing DFI values translate into higher fertilisation, pregnancy, or live-birth rates.

This distinction may help explain the heterogeneous literature on native-semen DFI and ART outcomes. Several meta-analyses have reported limited or inconsistent associations between elevated DFI and clinical pregnancy or live birth after IVF or ICSI [11,12,26], while other analyses have identified adverse associations that vary by ART modality [34]. A plausible explanation for this is that laboratory sperm preparation changes the cellular population to which the oocyte is exposed, thereby weakening the relationship between a measurement made in native semen and the DNA integrity of the selected sperm fraction.

Consistent with this interpretation, the predictive effect of DFI appears to vary across reproductive settings and may be attenuated when more stringent sperm selection is applied. Liu et al. found no statistically significant differences in fertilisation, cleavage, embryo, or clinical pregnancy rates between DFI subgroups in IVF/ICSI cycles, despite negative correlations between DFI and motility and survival [18]. Li et al. similarly reported associations with miscarriage and birth weight but not with fertilisation or pregnancy rates [35]. These observations do not render SDF testing clinically irrelevant; instead, they highlight that the timing and biological context of the measurement are likely important.

The present results also raise the possibility that the sperm-preparation method may influence the relationship between native DFI and the final fertilising population. Centrifugation-free, motility-based techniques produced the largest DFI reductions in our dataset, whereas DGC produced a smaller reduction. This pattern is compatible with previous concerns regarding centrifugation-associated reactive oxygen species generation [23,25], but clinical recommendations regarding method selection cannot be made from the current study because treatment allocation, laboratory indications, and reproductive outcomes were not analysed.

The findings also refine, rather than exclude, the so-called ‘iceberg’ hypothesis of SDF [36]. The marked difference between total and viable-gated DFI indicates that non-viable cells account for an important component of detectable DNA fragmentation in native semen. At the same time, the viable fraction retained a mean DFI of 11.27%, and previous studies have shown that viable SDF can discriminate fertile from subfertile men [28]. Thus, latent or cell-autonomous DNA damage within viable spermatozoa remains biologically plausible and potentially relevant to fertilisation and embryo development but not to clinical outcomes. Consistent with this framework, meta-analyses find no significant association between high native DFI and clinical pregnancy or live birth rates in IVF or ICSI [11,12,26]. If SDF testing is to retain clinical utility in the ART context, assessment should be performed on the processed, motile-enriched preparation—the fraction that will actually be used for fertilisation—rather than on the native ejaculate. Further studies incorporating reproductive outcomes and larger viability-gated cohorts are needed. This is underscored by recent umbrella-review and cohort evidence showing that native DFI shows weak or inconsistent associations with ART and early reproductive outcomes [37,38,39,40], reinforcing the case for viability-gated or post-preparation SDF assessment, with testicular sperm retrieval remaining a targeted option for the minority of men in whom elevated DFI persists despite adequate preparation [41,42,43,44].

Our framework, in which ART preparation systematically removes the non-viable, DNA-fragmented fraction, also has implications for the minority of patients in whom native DFI remains markedly elevated even after motility-based selection. In such cases, retrieval of testicular spermatozoa, which bypass post-testicular oxidative exposure during epididymal transit, has been increasingly investigated as an alternative source of gametes for ICSI. In an updated systematic review and meta-analysis, Zhao et al. found that testicular sperm carried substantially lower DFI than paired ejaculated sperm and that its use was associated with significantly higher clinical pregnancy and live birth rates and a lower miscarriage rate in men with high SDF [43]. A subsequent systematic review and meta-analysis by Cano-Extremera et al. corroborated these findings, reporting superior live birth rates and reduced miscarriage rates with testicular, compared with ejaculated, sperm in ICSI cycles from couples with high SDF [41]. Consistent with a clinically pragmatic approach, Ibis et al. [44] showed, in a cohort of 154 oligozoospermic couples with prior unsuccessful ICSI using ejaculated sperm, that switching to testicular sperm retrieval was associated with a meaningful proportion of subsequent live births, supporting testicular sperm retrieval as a rescue strategy after ICSI failure with elevated ejaculate DFI [43]. Together with the present findings, this literature suggests a two-tiered clinical approach: for the majority of patients, appropriate motility-based sperm preparation is sufficient to reduce the DNA-fragmented fraction to levels compatible with fertilisation, while testicular sperm retrieval may be considered a targeted option for the subset of men in whom elevated DFI persists despite adequate preparation, or after unexplained ICSI failure.

Several limitations should be considered when interpreting these results. First, the prospective viability-gated analysis was small (*n* = 11) and was conducted at a single centre. Second, the retrospective cohort included consecutive analyses only when all required variables, including DFI, were available, which may introduce selection effects and should not be interpreted as universal DFI testing of all clinic attendees. Third, reproductive outcomes were not analysed; consequently, the study can address associations between DFI, viability, motility, and sperm preparation but cannot directly establish predictive performance for pregnancy or live births. Fourth, apoptotic markers were not measured simultaneously with SDF in the same spermatozoa. Finally, the findings are based on SCSA methodology and may not be fully generalisable to SDF assays based on different analytical principles, such as TUNEL or Comet.

## 4. Materials and Methods

### 4.1. Patient Population and Sample Collection

Semen samples were collected by masturbation following a period of abstinence consistent with the WHO guidelines [1]. All analyses were initiated after complete liquefaction of the ejaculate and within 1 h of sample production.

### 4.2. Standard Semen Analysis

Sperm concentration was determined by Neubauer haemocytometer (Hawksley, Succexx, England) counting after fixation with a leukocyte-differential fixative (0.9% NaCl/3% H_2_O_2_/o-toluidine solution; 1:10 dilution); a minimum of 200 spermatozoa were counted in duplicate. Motility was assessed manually on fresh wet preparations. Fields were examined systematically, and each spermatozoon included in the count was classified as progressively motile, non-progressively motile, or immotile; total motility was calculated as the sum of progressive and non-progressive motility. A total of 100 spermatozoa was assessed per preparation. Vitality was defined as plasma-membrane integrity and was determined by eosin exclusion (5% eosin Y), based on the one-step eosin technique validated by Björndahl et al. [45]; live spermatozoa remained unstained (white), whereas membrane-compromised spermatozoa incorporated the dye (red), and 100 cells were counted per replicate. Nigrosin was not used because the protocol employed a direct wet-preparation eosin-exclusion endpoint rather than a permanent eosin–nigrosin contrast smear. These conventional semen-analysis procedures were performed according to the WHO Laboratory Manual for the Examination and Processing of Human Semen, 6th edition [1].

### 4.3. Sperm Preparation Techniques

Density gradient centrifugation (DGC): A two-layer discontinuous gradient was assembled by layering a 1 mL lower-density fraction, 1 mL upper-density fraction (Sage, CooperSurgical, Trumbull, CT, USA), and 2 mL neat semen in a 15 mL conical tube. Samples were centrifuged at 400× *g* for 12 min. The sperm pellet (0.2–0.3 mL) was aspirated, resuspended in 3 mL of washing medium (Sage, CooperSurgical), and centrifuged at 300× *g* for 10 min. The supernatant was carefully removed, leaving approximately 0.2 mL, and the sperm pellet was resuspended for post-preparation analysis.

Swim-up (SU): Two millilitres of washing medium was placed in a 15 mL conical tube, and the neat semen was layered beneath the medium. The tube was tilted to 45° to maximise the semen–medium interface area and incubated at 37 °C for 60 min. The upper fraction, enriched with actively migrating spermatozoa, was aspirated and transferred to a new tube for analysis.

Magnetic-activated cell sorting (MACS): After an initial DGC step (400× *g*, 12 min), the sperm pellet was resuspended in binding buffer (300× *g*, 4 min), and then incubated with annexin V-conjugated paramagnetic microbeads (~200 µL; 500 µL total volume in binding buffer; Miltenyi Biotec, Bergisch Gladbach, Germany) for 15 min at room temperature in the dark with gentle agitation every 5 min. The suspension was passed through a pre-equilibrated separation column positioned in a magnetic field; annexin V-positive (apoptotic) spermatozoa were retained on the column, while the apoptosis-negative eluate was collected, washed (300× *g*, 4 min), and resuspended in washing medium for analysis.

ZyMōt microfluidic chip (CHIP): A volume of 850 µL of neat semen was loaded into the inlet of the ZyMōt Fertile chip (CooperSurgical) via a 1 mL syringe. An additional 750 µL of washing medium was applied to the central separation area after priming the outlet with 50 µL of medium. The chip was incubated in a tri-gas incubator at 37 °C for 15 min. After incubation, 0.5 mL of the supernatant was aspirated from the outlet and transferred to a labelled conical tube for analysis.

### 4.4. Assessment of Sperm DNA Fragmentation—SCSA

SDF was determined by the sperm chromatin structure assay (SCSA) using the Cariad reagent system (Cariad Medical Technology, Zhuhai, China) and a MACSQuant 16 flow cytometer (Miltenyi Biotec). Spermatozoa were diluted to 1 × 10^6^ cells/mL in Reagent A (100 µL), followed by addition of 200 µL Reagent B with gentle mixing for 30 s, and then 600 µL Reagent C (Reagent C1 + C2; 1000:6 *v*/*v*), with 5 min of incubation in the dark. Samples were run at a medium flow rate using the blue (488 nm) laser; cells with intact double-stranded DNA emitted green fluorescence (525 nm) and cells with fragmented single-stranded DNA emitted red fluorescence (680 nm), and a minimum of 10,000 events were acquired per sample. Gating was performed sequentially on forward scatter (FSC) versus side scatter (SSC) to exclude sub-cellular debris, followed by FSC-height versus FSC-area measures to exclude doublets and aggregates; the remaining single-sperm population was then displayed on a red-versus-green fluorescence cytogram for DFI calculation. The DFI was calculated as follows: DFI (%) = [red fluorescence events/(red + green fluorescence events)] × 100. This ready-to-use kit is based on the originally published SCSA protocol of Evenson et al. [7].

For the native ejaculate versus viable-gated comparison (*n* = 11 paired samples), spermatozoa were first labelled with Viobility 405/452 Fixable Dye (Miltenyi Biotec; Cat. No. 130-109-816; excitation 405 nm/emission 452 nm), which stains cells with compromised membrane integrity: 1 µL Viobility per 1 × 10^6^ cells in 100 µL volume, incubated for 15 min at room temperature in the dark, followed by centrifugation (300× *g*, 10 min) and PBS washing. The SCSA reagent protocol was then applied as above. Within the sperm scatter gate, Viobility-positive events were classified as membrane-compromised/non-viable and Viobility-negative events were classified as viable. DFI was therefore determined both for the total sperm population and, using the Viobility-negative gate, for the viable-only subset within the same sample.

### 4.5. Retrospective Dataset

The retrospective analysis included 1394 consecutive semen analyses performed at the Dunamenti REK Tapolca Institute up to mid-2024 that fulfilled the prespecified inclusion criterion that sperm concentration, total motility, progressive motility, vitality, and DFI were all available. Thus, the cohort represents consecutive eligible analyses rather than an assertion that DFI was measured in every man attending the clinic. Samples were divided into two groups using the predefined DFI threshold: DFI < 25% and DFI > 25%.

### 4.6. Statistical Analysis

Data normality was assessed using the Shapiro–Wilk test (small *n*) or the Kolmogorov–Smirnov test (large *n*). Non-normally distributed variables were examined using the Kruskal–Wallis test; for multiple comparisons, Dunn’s test was applied. Normally distributed variables were analysed by one-way ANOVA, followed by Tukey’s test. Paired comparisons were analysed using the Wilcoxon matched-pairs signed-rank test. GraphPad Prism 8.0.1 software was used for all statistical analyses. All results were considered statistically significant at *p* < 0.05.

### 4.7. Ethical Approval

The study protocol was approved by the National Public Health and Medical Officer (11550-6/2022/EÜIG) and by the Regional Committee for Research Ethics, University of Pécs (8754-PTE/2021); the study was conducted in accordance with the Declaration of Helsinki.

## 5. Conclusions

In conclusion, our results indicate that (i) elevated DFI in native semen strongly correlates with reduced motility, vitality, and concentration based on the 1394-sample retrospective cohort, pointing to a shared biological origin for fragmentation and cell non-viability; (ii) in the paired viability-gated cohort, isolating the viable subpopulation lowered the mean DFI 3.5-fold, from 39.64% in the whole ejaculate to 11.27% in viable cells alone, showing that a large share of the fragmentation signal in native semen comes from cells that would never fertilise an oocyte; and (iii) every ART preparation method tested—density gradient centrifugation, swim-up, magnetic-activated cell sorting, and microfluidic selection—reduced DFI while enriching motile, viable spermatozoa, with the two centrifugation-independent techniques, the ZyMōt chip and swim-up, producing the largest reductions. Because fertilisation in ART is always carried out with a prepared, motile sperm fraction and never with native semen, a DFI value obtained before preparation describes cells that will not take part in fertilisation—a plausible reason why elevated native DFI so often fails to predict pregnancy or live births after IVF or ICSI. This does not argue against SDF testing as such, but against measuring it at the wrong stage: assessing the processed, motile-enriched sample would be more informative than assessing the raw ejaculate. Confirming this will require prospective studies that combine viability-gated DFI measurement with actual pregnancy and live-birth data in larger cohorts. In the smaller group of men in whom DFI remains high despite adequate preparation, testicular sperm retrieval offers a reasonable, targeted option.

## Data Availability

The raw data supporting the conclusions of this article will be made available by the authors on request.
